# Supplementary material for: Formation of Potential Heterotic Groups of Oat Using Variation at Microsatellite Loci
Source: Plants (Basel). 2021 Nov 15;10(11):2462. doi: 10.3390/plants10112462 (PMC8621079; doi:10.3390/plants10112462)
Supplement: Supplementary file 1 [file plants-10-02462-s001.zip › plants-1423690-supplementary.pdf]

Table S1. List of oat genotypes included in SSR analyses.

No.	Genotype	Origin	Releasing	Pedigree	Seed (glume colour, hull/naked)
1	Ábel	CZ	1993	Unnanem_7760 × Adam	naked
2	Adam	CZ	1988	Bento × 67-II	naked
3	Adler	S	2001	Seger × Von Lochowls – Gelbhafer	white
4	Ardo	CS	1990	Unnamed_7766 × KR-678	yellow
5	Argentina	I	1970	Argentina × Unknown	white
6	Aragon	D	1952	-	yellow
7	Arnold	D	1967	Lang-Dorflers-Weihenstephaner-Weishafers × Halle	white
8	Atego	CZ	2002	Gramena × Auron	yellow
9	Auron	CZ	1991	Unnamed_7766 × Veles	yellow
10	Auteuil		1997		brown
11	Aveia Peluda	P	1987	-	yellow
12	Avenuda (Jakub)	CZ	2001	KR-83-4057 × Semu581.1	naked
13	Avesta	A	1996	Selma × Sirene	brown
14	Azur	CZ	2001	Unnanmed_7776 × S325/81	yellow
15	BE 201 700	D	-	-	yellow
16	BE 202 299	D	-	-	yellow
17	BE 211 301g	D	-	-	yellow
18	Borka	D	1986	Selma × Erbgraf	yellow
19	Bug	RUS	1987	M9H61645 × Grundi	yellow
20	Coach	D	2001	-	yellow
21	Consul	A	-	-	yellow
22	Cyril	CZ	2001	Unnanmed_7839 × Zlaťák	yellow
23	Dagny	S	2001	-	white
24	Dalimil	CZ	2001	Unnanmed_7785 × Unnanmed_7831	yellow
25	Detvan	SK	2002	KR-88-8110 × KR 4057	naked
26	Eberhard	A		Dula × Flamingsnova	yellow
27	Edit	S	1990	-	white
28	Edmund	A	2001	Unnanmed_11609 × Panther	white
29	Euro	A	1995	-	yellow
30	Expander	A	1995	Dula × Flamingsnova	yellow
31	Expo	A	2001	Expander × Austral	yellow
32	Flämingslord	D	1997	STMG3054 × STRPBE-48-75D	white
33	Flämingsplus	D	1999	-	white
34	Flämingsprofi	D	2003	-	white
35	Flämingsstern	D	1971	Unnanmed_13917 × 1617	yellow
36	Flämingstrend	D	2005	-	yellow
37	Flipper	D	2000	-	white
38	Freddy	D	2001	-	white
39	Fusch	D	-	-	yellow
40	Gagybatory K Tajfajta	H	-	Landrace × Unknown	white
41	Gambo	NL	1989	Unnanmed_12728 × Marino	white
42	German	P	1994	-	yellow
43	Hannibal	D	1972	-	yellow
44	Hecht	D	1996	-	yellow
45	Hron (PS-81)	SK	-	-	yellow
46	Chantilly	F	2000	Adamo × 4720.18	white
47	Izák	CZ	1998	Unnanmed_7773 × Auron	naked
48	Jumbo	D	1995	Unnanmed_11632 × Fagot	yellow
49	Kanton	D	2001	-	yellow
50	Kubanskij	SU	-	-	yellow
51	Leo	D	2001	-	white
52	Lutz	D	1995	Unnanmed_11632 × Fagot	yellow
53	Maris Oberon	GB	1976	Manod × Unnanmed_9437	white
54	Markant	DK	-	-	white
55	Master	D	1995	-	white
56	Neklan	CZ	1998	Unnanmed_7766 × Auron	yellow
57	Nelson	D	-	Unnanmed_13932 × Unnanmed_13935	yellow
58	Neon	GB	1995	Unnanmed_7243 × 08659CN3-5N	naked

59	Noirine	F	1996	Creole × Major	brown
60	Pan	CS	1979	Diadem × Rigal	yellow
61	Pendek	NL	1954	Flamingsgold × Binder	white
62	Petra	S	1995	-	white
63	Pluco	NL	1986	-	white
64	Poncho	F	1996	-	white
65	PS-106	SK	-	-	naked
66	PS-90	SK	-	-	naked
67	Revisor	D	1996	Alfred × Dula	white
68	Salo	S	2002	-	white
69	Salomon	D	1994	Alfred × Unnanmed_11669	naked
70	Sanova AS 181 325	D	1987	-	white
71	Saul	CZ	2005	Unnanmed_16053 × KR5278	naked
72	Selma	S	1972	Palu × Saxo	white
73	Senator	A	-	-	yellow
74	Sirene	F	1979	-	brown
75	Stormont Sceptre	IRL	1972	-	white
76	Suomi	FIN	2001	-	white
77	SV-5	SK	-	-	naked
78	Šampionka	CZ	-	-	yellow
79	Tarra	A	1973	Flamingskrone × Peragold	yellow
80	Unisignum	D	1972	Regent (Bickelmann, 1989) × Phonix	yellow
81	Vendelin	SK	2007	Expander × Flamingsregent	yellow
82	Vilma	S	-	-	white
83	Vok	CZ	2002	Dalimil × Unnanmed_7786	yellow
84	Zlaťák	CZ	1988	Pan	yellow
85	Zvolen	SK	2006	Unnanmed_11688 × Auron	yellow

A – Austria; CS – Czechoslovakia; CZ – Czech Republic; D – Germany; DK – Denmark; F – France; FIN – Finland; GB – Great Britain; H –Hungary; I – Italy; IRL – Ireland; NL – Netherlands; P – Portugal; PL – Poland; RUS – Russia; S –Sweden; SK – Slovakia; SU – Soviet Union

Table S2. Primer sequences for oat microsatellite loci.

Code	Sequence	Repetition	Length (bp)	T_m (°C)	Reference
**AM1**	5′-GGATCCTCCACGCTGTTGA-3′<br>5′-CTCATCCGTATGGGCTTTA-3′	$(AG)_{21}(CAGAG)_6$	204	46	[57]
**AM4**	5′-GGTAAGGTTTCGAAGAGCAAAG-3′<br>5′-GGGCTATATCCATCCCTCAC-3′	$(AG)_{34}$	166	48	[57]
**AM6**	5′-AATGAAGAAACGGGTGAGGAAGTG-3′<br>5′-CCAGCCCAGTAGTTAGCCCATCT-3′	$(AG)_{20}$	209	52	[57]
**AM11**	5′-TCGTGGCAGAGAATCAAAGACAC-3′<br>5′-TGGGTGGAGGCAAAAACAAAAC	$(AG)_{12}(AAAG)_3$	225	49	[57]
**AM14**	5′-GTGGTGGGCACGGTATCA-3′<br>5′-TGGGTGGCGAAGCGAATC-3′	$(AC)_{21}$	133	48	[57]
**AM21**	5′-ACGTTGGTCTCGGGTTGG-3′<br>5′-AAATCCTTGACTTCGCTCTGA-3′	$(AT)_5..(AC)_5..(AC)_5$	210	46	[57]
**AM22**	5′-ATTGTATTTGTAGCCCCAGTT-3′C<br>5′-AAGAGCGACCCAGTTGTATG-3′	$(AC)_{22}$	138	46	[57]
**AM30**	5′-TGAAGATAGCCATGAGGAAC-3′<br>5′-GTGCAAATTGAGTTTCACG-3′	$(GAA)_{14}$	203	43	[57]
**AM31**	5′-GCAAAGGCCATATGGTGAGAA-3′<br>5′-CATAGGTTTGCCATTCGTGGT-3′	$(GAA)_{23}$	186	47	[57]
**AM83**	5′-CACTGCCATACATTCTGTCG-3′<br>5′-CCTCTACCGCAAAGGAAGAA-3′	$(AC)_{11}$	190	55	[58]

**AM84**	5′-GATAACAACTTGGATGCAACTGA-3′<br>5′-CAGGTTGACAAGGGAACGAT-3′	$(AC)_9$	163	55	[58]
**AM87**	5′-CACCAAGACGATTCGCTACA-3′<br>5′-CTTTTGCAAGACCTTCAAATCA-3′	$(AC)_{10}$	186	55	[58]
**AM91**	5′-GCGATTTACAACGACCTTATGA-3′<br>5′-CCAGTGCTTCTGATCTGGAAT-3′	$(AC)_{20}$	171	55	[58]
**AM92**	5′-CCGCACTATCGGCTTGTATC-3′<br>5′-GTGGGAATCGAGGGTAGAGC-3′	$(AC)_{13}$	130	60	[58]
**AM99**	5′-GCAATCATTCCAACTTGTTATGACACA-3′<br>5′-GATAGTGGAAGCGGCATCAACATC-3′	$(AC)_{12}$	169	58	[58]
**AM102**	5′-TGGTCAGCAAGCATCACAAT-3′<br>5′-TGTGCATGCATCTGTGCTTA-3′	$(AC)_9$	213	55	[58]
**AM104**	5′-AACAATGATGGGGATGGTGT-3′<br>5′-GTCGTGAGCAAGTTGAACCA-3′	$(AG)_{36}$	186	55	[58]
**AM112**	5′-AGCGGTGTAGGGGAAAGAGT-3′<br>5′-TTCTTGGTTTAGATGGGAGGA-3′	$(AG)_3(AC)_9(AT)_8$	234	55	[58]
**AM115**	5′-CGCAACTCTTCCTACTTTTTGTT-3′<br>5′-TGGCAAACTCCCTCGATTTA-3′	$(AC)_9$	214	55	[58]
**Rast1-4**	5′-CTTCTGCCCATGAAACCCTA-3′<br>5′-ACTCAGCACATGCACCCTC-3′	$(TAA)_3$	189	55	[59]

bp – base pair; T_m – melting temperature
